# Cold Plasma Systems and Their Application in Surface Treatments for Medicine

**DOI:** 10.3390/molecules26071903

**Published:** 2021-03-28

**Authors:** Francisco L. Tabares, Ita Junkar

**Affiliations:** 1 Laboratorio Nacional de Fusion, CIEMAT, Av Complutense 40, 28040 Madrid, Spain; 2 Department for Surface Engineering, Jožef Stefan Institute Jamova cesta 39, 1000 Ljubljana, Slovenia; ita.junkar@ijs.si

**Keywords:** plasmas, RONS, atmospheric plasmas, plasma material interactions, plasma medicine

## Abstract

In this paper, a review of cold plasma setups and the physical and chemical processes leading to the generation of active species is presented. The emphasis is given to the interaction of cold plasmas with materials used in medical applications, especially medical implants as well as live cells. An overview of the different kinds of plasmas and techniques used for generation of active species, which significantly alter the surface properties of biomaterials is presented. The elemental processes responsible for the observed changes in the physio-chemical properties of surfaces when exposed to plasma are described. Examples of ongoing research in the field are given to illustrate the state-of-the-art at the more conceptual level.

## 1. Introduction

In 1969, John R. Hollahan’s group experimentally demonstrated that with cold ammonia plasmas or mixtures of nitrogen and hydrogen, amino groups (–NH_2_) were produced, which, by adhering to the surface of different types of polymers, created materials compatible with blood [1]. Since then, the use of cold plasmas to optimize the interaction between biological systems and different types of materials has been investigated, with the ultimate goal of achieving biocompatible surfaces. Cold plasma treatment only affects the surface of the treated material. The physical, chemical, mechanical, electrical, and optical properties of the interior of the material are not altered by the cold plasma. On the other hand, the use of different acids and chemical solvents can damage the surface of many plastics and, if absorbed, affect the properties of the interior of the material.

Plasma is the predominant state of matter in the known universe (it is estimated that up to 99% of matter is plasma), although not on our planet, where the conditions of pressure and temperature make normal the states of matter—solid, liquid, and gas—that in global terms are exotic. It is enough to add energy to the solid (in the form of heat or electromagnetic radiation) for it to turn into a liquid state, from which gas is obtained through an additional supply of energy. If we continue adding energy to the gas, we will partially or totally ionize it, that is, we will remove electrons from the atoms or molecules that constitute it. In this way, we reach a new state of matter, plasma, made up of free electrons, atoms and molecules (electrically neutral particles), and ions. The energy needed to generate plasma can be supplied in several ways: through heat from a combustion process; through the interaction between laser radiation and a solid, a liquid, or a gas; or through electrical discharges in gases, in which free electrons take energy field and lose it through excitation and ionization processes of the atoms and molecules in the gas.

**Figure 1:**
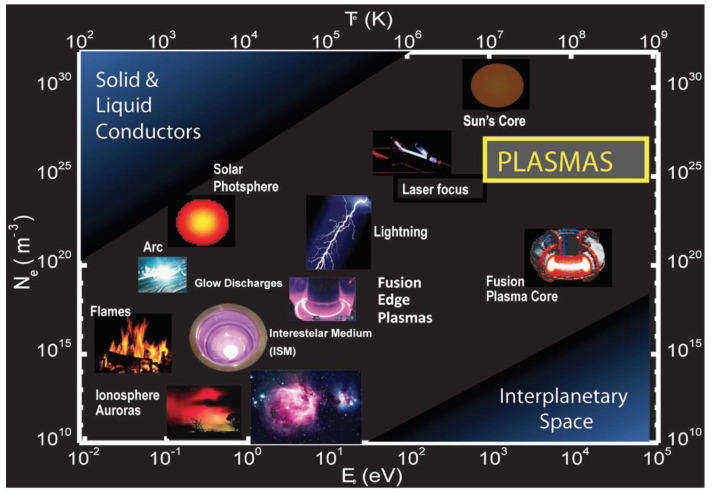
Overview of plasmas and the wide range of their microscopic parameters (electron energy and electron density). 1eV = 11,500 °C.

One of the peculiarities of plasmas is that they conduct electricity. On a macroscopic scale, plasmas are, however, electrically neutral, since the number of positive and negative charges is similar. Thus, the flame produced by burning candle wax in combination with oxygen from the air—a typical example of a plasma that is very little ionized—can conduct electricity. A graphical overview of the different kinds of plasmas according to their microscopic parameters (electron density and temperature) is displayed in Figure 1

The values of density and electronic temperature, two of the main parameters that characterize plasmas, cover a wide spectrum. Thus, the electron density varies between 1 electron/cm^3^ and 10^25^ electrons/cm^3^; that is, it even exceeds the concentration of electrons in metals. On the other hand, the average free path of the particles in a plasma, that is, the average distance covered before a particle collides with another particle in the plasma can range from tens of millions of kilometers to just a few microns. 

Classifying the diversity of types of plasmas that exist in nature or that can be generated artificially is not easy, since it is risky to choose isolated parameters that serve as criteria to establish the differences. Despite these difficulties, we can venture into a first classification of the types of plasmas, one that considers their thermal equilibrium, that is, whether or not the temperature or the average energy of the particles that make it up is the same for each type of particle. All particles have the same temperature (thermal equilibrium) for stellar interior plasma or for its terrestrial analogs, deuterium-tritium fusion plasmas and the impurities generated in experimental controlled nuclear fusion devices, like the JET and the ITER. These plasmas are also called hot or thermal plasmas since the temperature inside them reaches millions of degrees (10^7^ °C–10^9^ °C), the same for the electrons as for heavy species [2].

When the gas pressure is low or the electrical voltage applied in the discharge is high, the electrons in the plasma acquire, in the time between collisions with other plasma particles, kinetic energies higher than the energy associated with the random thermal movement of the neutral particles (atoms and molecules) of the plasma. We can then attribute some degree of thermal equilibrium deviation to plasmas, since electrons, ions, and neutral particles have different “temperatures” or average kinetic energies (in the case of non-Maxwellian distributions), rather common for plasmas produced at low pressure and with a small degree of ionization.

Nonthermal plasmas, also known as cold plasmas, are characterized by the fact that the temperature of heavy species (neutral particles and ions) is close to room temperature (25 °C–100 °C). Instead, the electronic temperature is much higher (between 5000 °C and 10^5^ °C). Cold plasmas usually occur at a low pressure (*p* < 133 mbar) in reactors with very different geometries. Such reactors generate plasmas through direct current, radio frequency, microwave, or pulsed discharge systems.

There are special types of cold plasmas, produced in the so-called corona and dielectric barrier discharges, that are generated at atmospheric pressure by using pulses between 10^–6^ s and 10^–9^ s. In these types of discharges, referred to as CAP (cold atmospheric plasma), the highly energetic electrons are produced that, due to the shortness of the pulses used, have little time to exchange energy with their surroundings. This kind of plasmas have found many applications in the field of plasma medicine, among others, and have led to off-the-shelf devices of increasing popularity.

The structure of this review is as follows. In Section 2, the fundamental concepts of plasma physics are introduced, together with the techniques applied to their production. In Section 3, the physical processes and the plasma chemistry behind the formation of active species of interest in biological applications involved in the interaction between plasmas and surfaces are reviewed. Finally, some examples from the recent literature are presented in Section 4.

## 2. Plasmas and Techniques for Their Generation

Here we shall describe the basic concepts of plasma physics and production techniques. Both topics are extensively described in many books and monographs, and a brief list of them is given in references [3,4,5,6,7,8,9,10,11,12,13].

Strictly speaking, a plasma is an ensemble of charged particles, either electrons or fully ionized atoms. However, there are very few examples of this kind of system and even in the core of a fusion reactor at 100.000 K chances of finding atoms or partially ionized impurities is not zero. Therefore, in a practical acceptation, partially ionized gases are also included in the term, being “gas discharge” the working definition for the cases of low ionization fraction of the gas. Under thermal equilibrium, the Saha–Boltzmann equation gives the distribution of ionic species in a plasma at a given temperature T [14]:

(1) $$\frac{ni+1}{ni}\;=\;\frac{2}{n_{e}}{\left(\frac{2\pi m_{e}}{h^{2}}\right)}^{3/2}{\left(kT\right)}^{3/2}\frac{\mu_{i+1}}{\mu_{i}}exp\left(\frac{-\xi_{i}}{kT}\right)$$

where *n_i_* and *n_i+1_* are the densities of ions with charge *i* and *i+1,* respectively, and n_e_ stands for the electron density. The ionization potential for the species “i” is represented by *ξ_i_* while its partition function is represented by μ_i_. The Boltzmann constant, *κ*, the electron mass, *m_e_*, and Plank constant, *h*, are also included in the equation. Note that this equation only works for a stationary plasma, i.e., with no transport of species. In fact, in a more realistic, dynamical situation, a further spreading of species takes place so that the observed distribution of charged states is less localized than that deduced from the local plasma temperature, even in thermal plasmas.

Plasmas are globally neutral and their electrical conductivity is significantly larger than any solid conductor. Again, for a fully ionized gas, its electrical transverse resistivity is given by the Spitzer formula [15].

Contrary to the solid conductors, an increase in the conductivity is expected at higher temperatures in plasmas. For the case of a partially ionized gas, on which electron-neutral rather than Coulomb collisions are responsible for, the plasma resistivity is given by [16],

(2) $$\;\;\;\rho {=}_{\;}\;m_{e}.\;\nu_{m}/e^{2}\;n_{e}$$

where *ν_m_* represents the electron-neutral collision frequency.

Global neutrality in the plasma volume is guaranteed by the Debye screening effect, but it doesn’t hold for distances below the so-called Debye length, *λ_D_*, approximately given by

(3) $$\;\;\lambda_{D}=\sqrt{\frac{k_{B\;}T_{e}\;\varepsilon_{0}}{n_{e\;}q_{e}^{2}}}$$

Typical values for cold plasmas are in the range of 0.1 mm and it plays a crucial role in the interaction between plasma and surfaces through formation of a sheath in the intermediate region (see next section). So, if a solid element is inserted into a plasma, a potential difference is established quickly between them, the solid resulting in a lower potential. The difference between this potential and the plasma own potential is known as the floating potential:

(4) $$\Delta V=\;\left(k_{B}\;T_{e}/e\right)\left(2.8+0.5\ln\mu_{i}\right)$$

where μ_i_ is the mass of the plasma ion referred to that of hydrogen. The reason for that is the much higher mobility of electrons than that of ions, leading to a fast, negative charging of the solid, and eventually, to the suppression of the flux of (thermal) electrons and acceleration of the plasma ions toward the material. As a consequence, the electric filed generated in the boundary between the plasma and its solid container is quite high, in the range of kV/cm, and may have its own effect on living organisms, while in solid surfaces, it may trigger sparks or arcing.

The most commonly used method of generating and sustaining a low-temperature plasma for technological and technical application is by applying an electric field to a neutral gas. Any volume of a neutral gas always contains a few electrons and ions that are formed, for example, as the result of the interaction of cosmic rays or radioactive radiation with the gas. These free charge carriers are accelerated by the electric field and new charged particles may be created when these charge carriers collide with atoms and molecules in the gas or with the surfaces of the electrodes. This leads to an avalanche of charged particles that is eventually balanced by charge carrier losses, so that a steady-state plasma develops.

**Figure 2:**
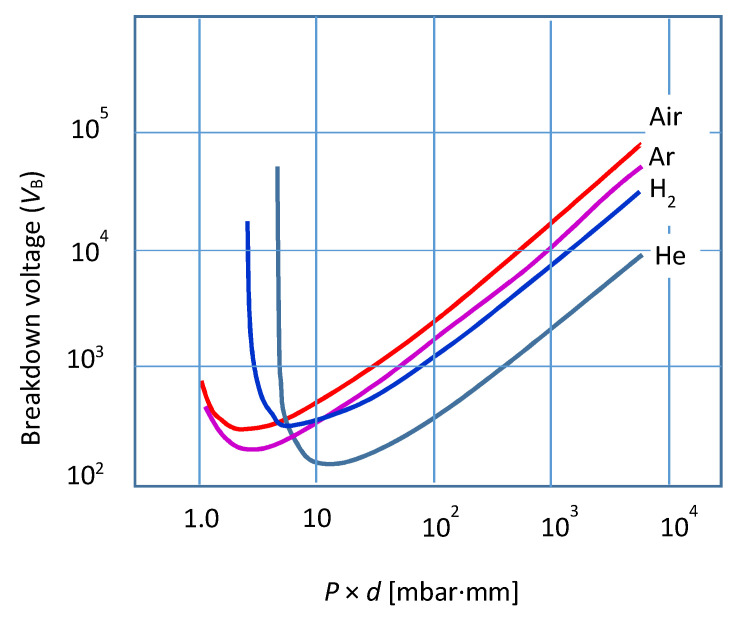
Variation in breakdown voltage, *V*_B_, with the product *P* × *d*, in electrical discharges for several gases (see the text).

The electrical break-down of the gas is obtained under specific conditions of pressure, voltage, and electrode secondary electron emission, as reflected by the Paschen expression:

(5) $$V_{B}=\;\frac{B\times \;P\times \;d}{C+\ln\left(p\times d\right)}$$

where *B* and *C* are constant parameters depending on the gas mixture and the cathode material. The law is fulfilled not only in DC but also in a wide frequency range of alternating current (AC) discharges. Figure 2 shows Equation (5) for several gases [13] (Section 2).

**Figure 3:**
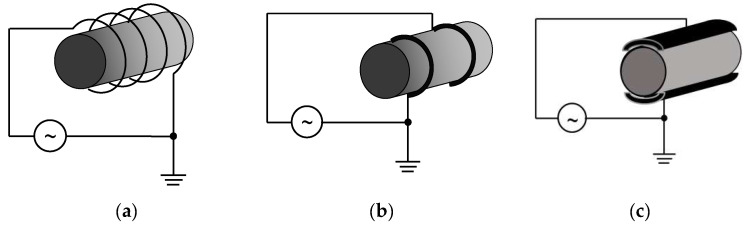
Scheme of RF plasmas. From left to right: (**a**) inductive, (**b**) capacitive with annular electrodes, and (**c**) with parallel electrodes.

Historically, the most studied plasmas were those generated with direct current (DC) discharges. The development of electric power supplies able to provide alternative or pulsed voltages for various applications (energy transport, communication (radio) technology, radars, etc.,) impacted on the plasma field and currently plasmas are generated also by such voltages. Therefore, beside DC discharges, a wide variety of discharges are utilized nowadays: i) low frequency discharges (a few Hz up to kHz); ii) high frequency discharges (kHz to hundreds of kHz); iii) radiofrequency discharges (in the MHz range, typically tens of MHz—radio waves); iv) microwave discharges (GHz range—compared with the microwave oven—2.45 GHz). As an example, Figure 3 shows different schemes for RF plasma generation. In addition, pulsed electrical discharges (negative or positive pulses with the duration from ms to ns) and various repetition rates should be added in this enumeration; vi) laser generated discharges are obtained by focalized pulsed plasma beams—optical breakdown at 10^14^ Hz—these are pulsed plasmas, with pulse durations in the ns or ps range (using ns and ps lasers).

**Figure 4:**
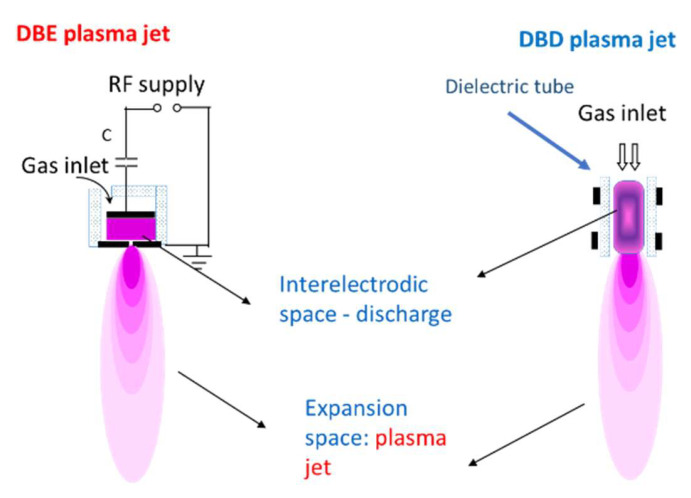
Scheme of a dielectric barrier discharge (DBD) and a dielectric barrier electrode (DBE) and the extracted plasma beam from Ref [13], Chapter 7.

For some applications, requiring operation under atmospheric pressure and contact between the plasma and the target, as in many medical uses, a beam of cold plasma is extracted from the source. Ignition of the discharge at high pressure, depending on the *p.d* product (Paschen law), calls for a very small interelectrode distance, typically less than 1 mm. Figure 4 shows two examples of Dielectric Barrier discharges used in these applications [13] .

## 3. Interaction between Plasmas and Surfaces. Physical and Chemical Processes

The application of plasma technology to biomedicine may result from several conceptions. Immersion of biomaterials in a plasma reactor, typically at low pressure, is most convenient for surface modifications like coating or patterning, including enhancement of hydrophily or roughening of the surface. Plasma jets (pencils), generated from cold atmospheric plasmas (CAP, Figure 4), are suitable for in situ treatment of live tissue [17], as in skin [18], teeth [19,20], or chronic wounds [21]. CAP may also be used to modify the reactive species present in an aqueous solution [22] through plasma–liquid interactions. In any case, both physical and chemical interactions are behind the observed effects.

In a cold plasma, a large variety of species can be found. In addition to charged particles, atoms and radicals, both in ground, excited and metastable states, as well as molecules and photons in a wide range of wavelengths are present. As mentioned above, an electric filed is generated in the boundary between the plasma and the solid, being maximum under the conditions of DC low pressure discharges. Ions created in the plasma volume by electron impact processes are accelerated toward the solid with energies given by their temperature and the sheath potential

E = V_plasma_ + 2kT + Z.V_sheath_ (6)

with Z being the charge state of the plasma ion, and T a common temperature for electrons and ions. For a hydrogen plasma E_kin_ ≈ 5kT if ions are thermalized with electrons, which is not the case for cold plasmas as stated before. At low Ti, E_kin_ ≈ V_plasma_ + Z.V_seath_ ≈ V_plasma_ + 3T_e_. In a DC GD, V_plasma_ is basically the applied voltage between electrodes but in RF o ECRH plasmas it is given by the so-called self-bias voltage, a few times Te and depending on electrode configuration, among other factors. Depending on the pressure, ions will gain the full kinetic energy given in Equation (6) (collisionless sheath) or will undergo many collisions with the background gas in their way to the cathode and lead to its heating. This is the case of CAP, characterized by weak ion–surface interactions in the absence of external DC bias.

The effect that high energetic ions have on material surfaces is the subject of a score of studies and excellent books, especially in the field of fusion plasmas, are available in the topic [23,24,25]. Ballistic effects account for sputtering and implantation, the first leading to morphological changes on the surface (roughness) and the latter followed by thermal diffusion into the bulk and surface recombination or trapping in defects. On the other hand, secondary electron emission from the bombarded surface plays a key role in the initiation and maintenance of the plasma. The resulting, low-energy electrons are accelerated in the cathode sheath and undergo elastic inelastic collisions with the gas, triggering the ionization, dissociation, and excitation of the atoms and molecules in it (see below). Sputtered particles may result in their ground or excited electronic state or as ions.

When low pressure plasmas are used for surface coating, high energies of the film precursor are sought for good adhesion of the film [13] (Section 2). Changes in surface roughness alter the wetting properties of the substrate and this is used for hydrophobic to the hydrophilic conversion of the exposed surface. When the plasma species is chemically reactive with the substrate, a different kind of process, tagged as chemical sputtering, takes place. This is the case for example of carbon or silicon-containing materials exposed to H plasmas and ion energy and substrate temperature [25] and molecules and radicals are created in the interaction.

Plasma chemistry is indeed a complex subject due to a large number of species and associated chemical reactions which may be produced after the primary electrical breakdown of the gas by the accelerated electrons. Aside from the chemical products of the promoted reactions, several elementary processes are behind the generation of the different species characteristic of chemically active, low temperature plasmas:

### 3.1. Electron Impact Ionization: e^−^ + A → A^+^ + 2e^−^

Electron impact ionization is the fundamental process by which plasma itself is created and sustained by electron multiplication. The electron energy has to overcome a threshold set by the ionization potential, E_iz_, of the gas species, A, which for cold plasmas, with Te < E_iz_, means that only a small fraction of electrons, in the high E tail of the EEDF (electron energy distribution function), are available for this process. For DC plasmas at low pressure, secondary electrons accelerated by the cathode sheath enter the plasma volume and undergo elastic and inelastic collisions leading to their thermalization with thermal electrons. In AC discharges, the alternating electric field accelerate electrons periodically changing their direction within the plasma, largely enhancing their collisionality. Depending on the frequency, ions may or may not be able to reach the solid surfaces, leading the secondary electron production as in DC discharges, and the amplitude of the oscillation decreases as the pressure is increased.

This process has a symmetric version, particularly relevant for low temperature, high pressure plasmas as in CAP, electron recombination:

*e* + *A*^+^ → A^*^ → *A* + *hν* (7)

The resulting photons may play an important role in the application of the plasma, as described below. Also, for very specific cases as highly electronegative species and selected electron energies, the electron attachment process, e^−^ + A → A^−^, may compete with ionization.

### 3.2. Electron Impact Excitation: e^−^ + A → A^*^ + e^−^

The process is analogous to the previous one, this time leading to the formation of an electronically excited state of the gas species, A*. This species may decay quickly (τ < 1 μs) by electronic emission in the form of light or become metastable if no emission is allowed by quantum mechanical laws (dipole transition). The characteristic photon emission of the plasma driven by electron excitation represents a fingerprint of the contained species and its analysis provides one of the most powerful diagnostic in plasma chemistry, OES (optical emission spectroscopy). Furthermore, these photons may lead to the desorption of gas from the reactor walls as well as irradiation of any element attached to them, which for UV wavelengths (tens to hundreds of nm) represents a potential source of bactericide. Metastable states show a rather high ionization probability. Obviously, a lower threshold exists in this case and only at high electron densities this kind of excitation may lead to the so-called step-like ionization, requiring two electron collisions with energies below E_iz_. In addition to that, metastable species, having a large stored potential energy, may play a key role in promoting endothermic chemical reactions or producing ion species through the Penning processes, A^*^ + B → A + B^+^ +e^−^, of high relevance in CAP.

### 3.3. Electron Impact Dissociation: e^−^ + AB → A+ B + e^−^

Perhaps this is the elemental process most relevant for plasma chemistry. It accounts for the formation of highly reactive atoms and radicals from otherwise inert species. Furthermore, the dissociation products, A,B, may result in excited, metastable, or even ionic states, thus triggering a set of reactions not observed in pure thermal chemistry. As a possible intermediate state, a vibrational or rotational excited molecule, AB (v)*, or molecular ion, AB^+^, may be involved in the collision, so that the electronic, kinetic, vibrational, and rotational temperatures of the gas may significantly differ, as experimentally documented. The relative dissociation efficiency of different plasmas relative to their ionization capabilities is an important parameter to be considered in the production of atomic species, with inductive RF plasmas showing the best performance [13] (Section 4)

### 3.4. Ion processes

Charge exchange between atoms and ions (or between different ionic species) is a well-known, important process taking place in high ion-temperature plasmas, as in fusion research. Positive ions and atoms may exchange charge states after transfer of a valence electron from the atom to the ion:

*A*^+^ + *B* → *A* + *B*^+^

In cold plasmas, charge transfer between molecular species plays an important role in its chemistry, driven by the different proton affinities of the plasma components.

All in all, gas phase and surface chemistry reactions have to be included in a realistic model of the plasma and its observed effects on exposed samples. These reactions can be shorted out based on the nature of the implied species. In addition to the elementary processes already described, the following ones should be included:

Homogenous (gas phase)

- Ion-ion
- Ion-molecule
- Radical-radical
- Radical-molecule
- Molecule-molecule

where the electronic (metastable) and internal excitation (vibrational, rotational) of the reactants are treated as different species.

Heterogeneous (gas-surface)

- Surface absorption
- Surface recombination
- Diffusion
- Surface desorption
- Surface reactions
- Surface de-excitation(quenching)
- Film formation

Obviously, the relative role that each elemental process plays in the yield of active species production may differ by orders of magnitude and some short of simplification of the otherwise untreatable system of kinetic equations is usually performed.

## 4. Plasma Treatment of Medical Materials

Although the very state-of-the-art of the topic is represented by the kind of research articles included in this special issue, in order to illustrate the practical applications of cold plasmas in the field of biomedicine under the light of the plasma physio-chemistry, two classical examples are addressed herein: enhancement of the wetting properties and biocompatibility of medical surfaces and generation of active radicals as bactericides, which are more commonly used for direct treatment of bacterial infections. While surface treatments are mainly (but not exclusively [11,13]) carried out under vacuum conditions, thus profiting from the high energies of the ionic species, active radicals for in situ treatments are produced at atmospheric pressure (CAP).

**Figure 5:**
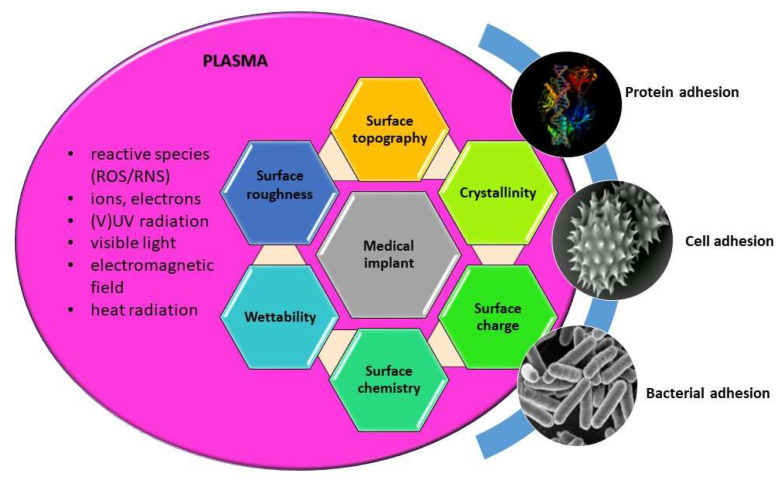
Interaction of gaseous plasma with medical surface and its influence on surface properties which further dictate biological response (interaction of proteins, adhesion of cells, bacterial adhesion, and biofilm formation).

Thus the use of low pressure plasma in medicine is basically aimed at the modification of surfaces, as it is well-known that biological response is mainly governed by surface properties of materials, such as wettability, chemistry, morphology (nanotopography), crystallinity, as well as surface charge. All these surface features can be altered by plasma treatment, as already discussed in previous sections. Schematic presentation on induced surface changes and their influence on biological interactions; adhesion and conformation of proteins, adhesion and proliferation of cells, and bacterial adhesion are schematically presented in Figure 5. By appropriately adjusting the discharge and plasma parameters desired modification of material surface used for a specific medical application can be achieved.

**Figure 6:**
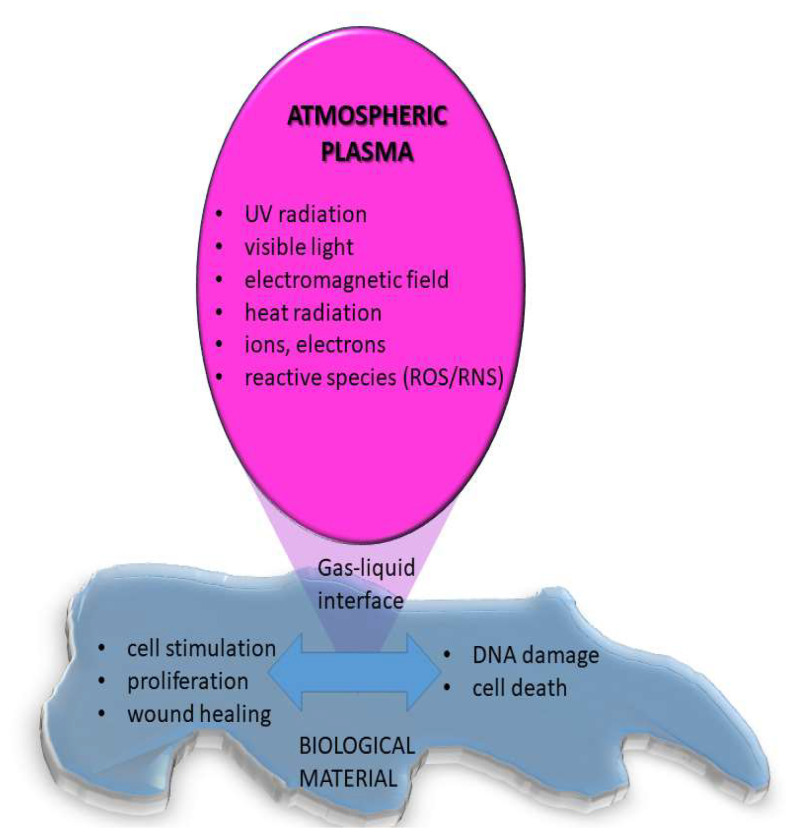
Schematic presentation of atmospheric pressure plasma interacting with biological material.

On the other hand, when atmospheric pressure plasma is used for medical applications, the interaction of plasma species with living organisms or liquids is addressed [26] as schematically displayed in Figure 6. In this case, the plasma may stimulate cell adhesion and proliferation or can cause cell death. It was shown that plasma can selectively kill cancer cells but not healthy cells, mainly due to RONs [22]. Treatment of bacterial infections, especially in case of chronic wounds was also shown to have significant success and it is already medically approved and used in praxis [21]. Results of a randomized clinical trial show that treatment of diabetic foot ulcers by CAP (kINPen Med; neoplast tools GmbH) reduced wound size, clinical infections, and microbial load compared to the initial state [27]. By optimizing the treatment conditions it is possible to achieve a desired biological response, although the exact mechanisms of interactions mainly based on redox reactions are not fully known and understood.

### 4.1. Enhanced Surface Hydrophilicity and Cell Adhesion

The adhesion of cells to surfaces of clinical application (especially polymers) requires the modification of their wetting characteristics. When using plasma techniques, this is achieved through a series of interrelated processes on which physical and chemical interactions play an important role. Even inert gases can modify the surface chemistry through ablation, crosslinking, and surface activation [25].

In the ablation process, the bombardment of the polymer surface by free radicals, electrons, ions and radiation breaks the covalent bonds of the polymer backbone, resulting in lower-molecular-weight polymer chains. As long molecular components become shorter, the volatile oligomer and monomer by-products vaporize off (ablate) and are swept away with the exhaust.

Crosslinking in plasma is believed to be highly correlated with vacuum ultraviolet (VUV) radiation and is usually achieved with an inert process gas (Ar or He), which has quite intense emissions. However, the mechanisms here are not very clear as the synergistic effects of the plasma species may also play an important role. It was shown in a study by Tajima et al. [28] that the extent of crosslinking of PE polymer by inductively coupled Ar plasma was due to the simultaneous effects of uncharged particles and VUV, while the effects of ion bombardment were secondary. According to a study by Luque-Agudo et al. [29] treatment of biomedical relevant polylactic acid (PLA) films by oxygen and argon plasma resulted in different hydrophobic recovery. It is well-known that after plasma treatment polymeric surfaces tend to age to their more favorable energetic state and become more hydrophobic. In the present study the initial value of water contact angle (WCA) was about 81°, after oxygen and argon plasma it was reduced to 57° and 51°, respectively. However, after 12 weeks of aging in the air the WCA increased to about 67° and 40° for oxygen and argon plasma, respectively. This was correlated with higher crosslinking of polymer chains, as well as the fragmentation into low molecular weight moieties (ToF-SIMS analysis) after argon plasma treatment. Also, recently, Jaritz et al. [30] performed a comparative study of the effect of argon and oxygen plasmas and that of their isolated radiations on the adhesion of organosilicon coatings on polypropylene. It was shown that the same maximum bond strength enhancement can be reached by pretreating the polypropylene surface either with pulsed oxygen plasma or with only the UV radiation from this oxygen plasma. Interestingly, for argon plasma no significant influence of its UV radiation on the substrate could be observed. Two main differences between plasma or just radiation exposure should be kept in mind, although when comparative studies of this kind are addressed. First, the dose of active species reaching the surface when directly exposed to a plasma is orders of magnitude larger than the corresponding photon fluxes achieved by conventional UV lamps. Second, while the range of interaction of ions and radicals is very shallow (few nm) at the energies involved, UV photons can penetrate up to one micron into the material [31]. The different ranges of interaction, among other factors, maybe behind the observed synergetic effects, difficult to pinpoint in isolated experiments.

Anyhow, the bond breaking occurs on the polymer surface and, in the absence of free-radical scavengers, it can form a bond with a nearby free radical on a different chain (crosslink). When reactive species are mixed with the inert carrier, cross-linking of the new-born radicals competes with the formation of the reaction products with the reactive component and a new set of covalent bonds arises.

The bond breaking occurs on the polymer surface but, since there are no free-radical scavengers, it can form a bond with a nearby free radical on a different chain (crosslink). When reactive species are mixed with the inert carrier, cross-linking of the new-borne radicals competes with the formation of the reaction products with the reactive component and a new set of covalent bonds arises.

Activation is a process where surface polymer functional groups are replaced with different atoms or chemical groups from the plasma. As with ablation, surface exposure to energetic species abstracts hydrogen or breaks the backbone of the polymer, creating free radicals. Besides, plasma contains very high-energy UV radiation. This UV energy creates additional free radicals on the polymer surface. Free radicals, which are thermodynamically unstable, quickly react with the polymer backbone itself or with other free-radical species present at the surface to form stable covalently bonded atoms or more complex groups. This is known as surface functionalization, which can be, from the medical application point of view, used for direct interaction with biological material [32] or used to improve adhesion of various types of bioactive coatings [33,34,35].

Although the kind of energetic process involved in bond-breaking and sputtering may favor the use of low-pressure plasmas, it has been shown [36] that the same effects can be achieved through the use of atmospheric pressure plasmas, thus easing the practical implementation of the adhesion-enhancing plasma technologies.

From a pure physical point of view, surface roughness is enhanced through the sputtering and the erosion mechanisms just described. So, the effective area of the substrate interacting with the biological specimen or with the atmosphere is enlarged. In this process, the microstructure of the polymer or substrate, in general, plays a critical point. Thus, for example, Junkar [37] demonstrated that the degree of crystallization of the polymer has a direct impact on its biocompatibility when treated by a nitrogen or oxygen RF plasma, obtaining better results on amorphous samples. Thus surface nanostructuring can also be one of the important surface features which alone or in combination with other surface features improves the biological response of medical materials.

**Figure 7:**
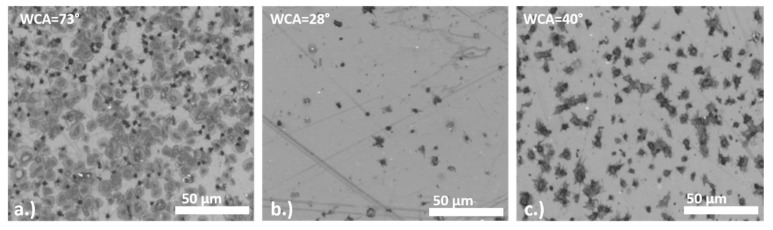
Interaction of platelets incubated with PET surface; (**a**) untreated, (**b**) oxygen plasma treated, (**c**) oxygen plasma treated after 2 weeks of storage in air [38].

One common issue found in surface activation by plasma of polymers is the limited lifetime of the positive results obtained, eventually resuming into its initial, low activation state (aging effect). As surfaces may change its hydrophilic character already after few hours of plasma treatment (depending on the type of polymer and plasma treatment used) this should be considered, as wettability may significantly influence the biological response. For example, changes in wettability of polyethylene terephthalate (PET) surface after RF oxygen plasma treatment (power 200W, pressure 75 Pa, treatment time 30 s) significantly influence the biological response [38]. The surfaces were incubated with whole blood immediately after plasma treatment and after 1 weeks of storage in the air. In the case of blood connecting medical devices, platelet adhesion should be prevented to reduce the risk of thrombosis, thus a lower amount of platelets in non-activated states (more round state) is desired. It can be observed from Figure 7 that platelet adhesion is highly correlated with surface wettability. The interaction of platelets with the surfaces analyzed with optical microscopy is presented in Figure 7. Platelets readily interact with untreated PET surface where WCA is about 72°, while significantly lower platelet adhesion is observed for the case of oxygen plasma treated surface, where WCA is about 28°. After aging in air for 2 weeks the increase in platelet adhesion is again observed, in this case WCA is about 40°. However, it should be emphasized that also other factors, like reorientation of functional groups from the surface into the bulk of the polymer due to aging may play a role in the cell–surface interactions, as well as the type of newly formed functional groups. Moreover, the study by I. Junkar et al. [31] already showed that treatment of PET in nitrogen plasma provides similar surface wettability compared to oxygen plasma treated surfaces, however, platelet interactions are different. On nitrogen plasma-treated surface platelets strongly adhere (similar as to the untreated surface), which supports the fact that wettability is not the only parameter that should be considered.

**Figure 8:**
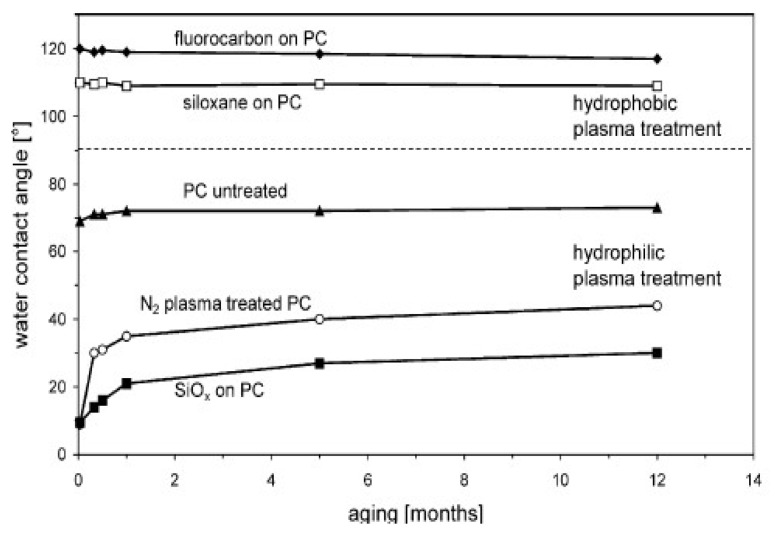
Aging of untreated, plasma treated and coated PC sheets. The polycarbonate surface can be rendered hydrophobic or hydrophilic [36]

In this respect, in order to prevent surface-induced aging, a very significant improvement has been achieved by thin film coating of the polymer. Thus, for example, Hegemann et al. [36] showed that the deposition of ultra-thin layers by plasma enables the long-lasting adjustment of wetting properties, using siloxane-based or fluorocarbon films, and the reduction of the friction coefficient, applying siloxane or a-C:H coatings, as shown in Figure 8. Vast number of studies used plasma pretreatment step in order to enhance the adhesion of bioactive coatings on highly non-reactive polymeric medical materials like polyethylene (PE) [39], polycarbonate-urethane (PCU) [35], polylactic acid (PLA) [40], polytetrafluoroethylene (PTFE) [41,42].

### 4.2. Plasma Generation of RONs for Medical Treatment

Plasma has emerged as anti-cancer therapeutic agent given its cancer-cell-apoptotic action. In particular, cancer-cell-selective killing by air or Ar plasma has been observed, although exactly how and why cancer cells are sensitive to plasma remains unknown. Although the biologically effective species generated from plasma and its cellular targets remain unknown, several lines of evidence link reactive oxygen/nitrogen species (ROS/RNS) to its biological effects, and targeting cancer cells through ROS-mediated mechanisms has become an attractive strategy for the effective and selective cancer treatment by exploiting the aberrant redox characteristics of cancer cells. It has been postulated [43] that ROS/RNS induced by air or Ar plasma effectively targets cancer cells via mitochondrial dysfunction and activation of oxidative stress signaling pathways [43,44,45]. However, this issue is highly complex as there are also other indications like immune system modulation, which may also induce cell death.

Complex chemistry is triggered when an electrical discharge is produced in air at atmospheric pressures. The initially produced molecular and atomic ions of the air components quickly undergo collisional processes releasing energy, thus heating the gas, and initiating a series of chain reactions ultimately leading to the production of the active agents. Commonly observed chemical species at significant concentrations include ozone (O_3_), singlet delta oxygen (O_2_,a^1^Δ_g_), atomic nitrogen (N), hydroxyl radical (OH), various nitrogen oxides (e.g., NO, NO_2_, and N_2_O), hydrogen peroxide (H_2_O_2_) and nitric and nitrous acid (HNO_3_, HNO_2_), among others. In some cases, these species are similar or identical to known RONS of importance in biology [43].

**Figure 9:**
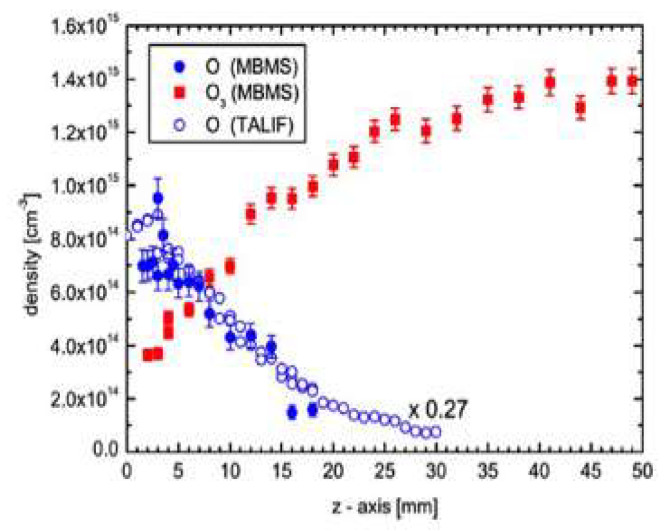
The measured density of O and O_3_ as a function of distance from the tip of a He/O_2_ plasma jet, using two different methods: molecular beam mass spectrometry (MBMS) and two-photon absorption laser-induced fluorescence (TALIF) [46].

An overwhelming effort has been devoted to the simulation of these highly complex systems [6], although only through targeted experiments it is possible to disentangle the individual impact of selected candidates. As an example, Figure 9 shows the measured atomic oxygen and ozone concentration distribution for a He/O_2_ plasma (right) [46]. The results closely follow the predicted distribution of active O species along the plasma jet for an Ar/O2 plasma [47]. So, although a different main gas is used, very similar results are obtained, as expected from the lack of chemical effects by noble gases. Also, according to the model predictions, a very fast decay of charged particle density takes place in the jet, and metastable O_2_ molecules together with ozone and nitric oxide are the only species surviving one centimeter away from the nozzle [47].

In addition to electron-mediated processes, and depending on the gas flow, temperature effects may play a role in the generation of active agents. Very recently, Cejas et al. [48] developed a model of a stationary glow-type discharge in atmospheric-pressure air operated in high-gas-temperature regimes (1000 K < Tg < 6000 K), focusing on the role of associative ionization reactions involving N (2D,2P) excited atoms. The kinetic model included processes involving positive (i.e., NO^+^, N_2_^+^, O_2_^+^, and O^+^) and negative ions (O^−^, O_2_^−^, and O_3_^−^), neutral species (i.e., N_2_ in the ground and vibrational and electronic excited states, N(2D), N(4S), N(2P), O(3P),O(1D), O(1S), O_2_, and NO), and electrons (e) which, together with the corresponding chemical reactions accounts for more than one hundred elemental processes in the gas phase. As a distinctive feature, the model incorporated an exoergic associative ionization reaction with the participation of N(2P) atoms and a near-threshold reaction with the participation of N(2D) atoms with a low activation barrier of 0.38 eV which can be described as:

O(3 P) + N(2 P) → NO^+^ + e

O(3 P) + N(2 D) → NO^+^ + e

The results of the calculations suggested a strong impact of the electronically excited states of reactants on associative ionization reactions in atomic collisions in hot air.

**Figure 10:**
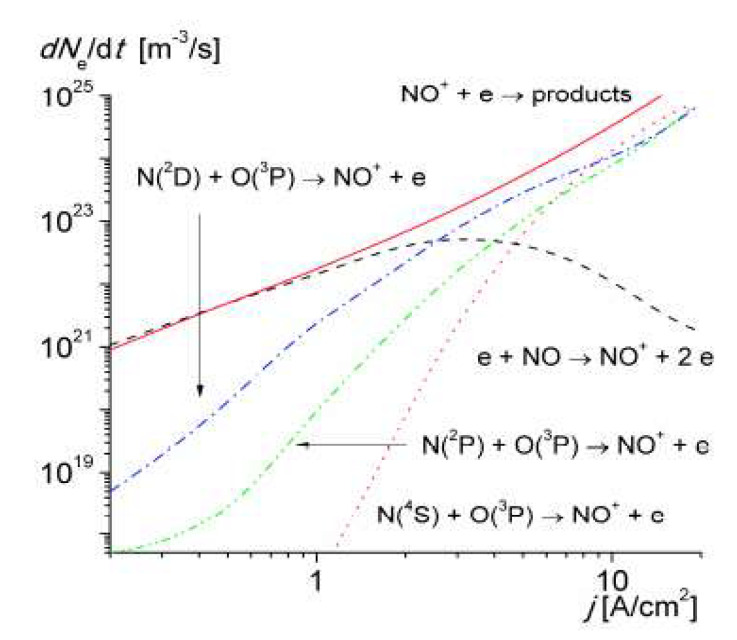
The rates of electron production and loss via various mechanisms versus the discharge current density calculated for R = 1.0 mm [47].

It was shown that the near-threshold associative ionization reaction involving N(2 D) atoms progressively replaced the ionization of NO molecules by electron impact at Tg > 2500 K (corresponding to a current density of > 1A/cm^2^), becoming the main ionization mechanism in air up to 4000–4500 K. Figure 10 shows the relative contribution of associative ionization to the electron production rate in the discharge.

Finally, it should be noted that models become exponentially expensive in terms of computer time as the number of species included in them increases. Very recently, He et al. [49] modeled the kinetics of a micro-scaled atmospheric pressure plasma jet (μAPPJ) of He/N_2_/O_2_ by using a 0-D model for the chemistry and plug flow for the gas transport (pseudo 1-D model). A total of 41 species and 516 reactions were initially considered. However, inclusion of vibrational levels of the molecular species (deemed necessary to take into account their contribution to the high energy tail of the EEDF in N_2_-containing plasmas [50] boosted the system to 138 species and 11733 elemental reactions. Even so, matching the experimental results on nitric oxide production by the plasma required the inclusion of metastable N(D) reactions and some tuning of the rate constants involved in its generation.

In conclusion, and is spite of the tremendous efforts made in the field, the complexity of the systems involved in the therapeutic applications of CAP research makes it difficult to predict the healing properties of these plasmas, and a rather empirical approach to the topic prevails so far. Very well-defined experiments assisted by modeling may render a deeper understanding of this highly promising subject.

## 5. Conclusions

The main mechanisms of the physical and chemical processes leading to surface modifications by cold plasmas are discussed under the light of its application in medicine. Interaction of plasma species with medical material enables modification of surface properties, which significantly influence the biological response. However, the stability of modification is not long-lasting, thus in many cases, additional coating techniques are used to overcome this drawback. In the case of atmospheric pressure plasma, its use is not limited only to medical materials as live tissues or even liquids can be treated. This highly prospective field of research has already shown significant success in cancer therapy, healing of chronic wounds, and dental applications. However complex mechanisms of plasma interaction with cells or liquids are still poorly understood and further studies in this direction have to be conducted to optimize the plasma treatment conditions for the specific application. It can be speculated that plasma treatment technologies for medical application will exponentially grow in the forthcoming years, as this field of research is still in its infancy and there are still many unknowns.
